# Pluronic F68 Micelles as Carriers for an Anti-Inflammatory Drug: A Rheological and Scattering Investigation

**DOI:** 10.1021/acs.langmuir.3c03682

**Published:** 2024-01-03

**Authors:** Nicola Antonio Di Spirito, Nino Grizzuti, Viviane Lutz-Bueno, Gaia Urciuoli, Finizia Auriemma, Rossana Pasquino

**Affiliations:** † DICMaPI, Università degli Studi di Napoli Federico II, P. le Tecchio 80, 80125 Napoli, Italy; ‡ Laboratory for Neutron Scattering & Imaging, Paul Scherrer Institute, CH-5232 Villigen PSI, Switzerland; § Dipartimento di Scienze Chimiche, Università di Napoli “Federico II”, Complesso Monte S. Angelo, via Cintia, 80126 Napoli, Italy

## Abstract

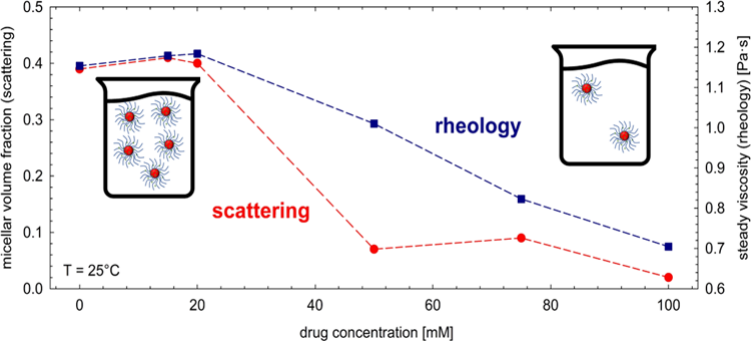

Age-long ambition of medical scientists has always been advancement in healthcare and therapeutic medicine. Biomedical research indeed claims paramount importance in nanomedicine and drug delivery, and the development of biocompatible storage structures for delivering drugs stands at the heart of emerging scientific works. The delivery of drugs into the human body is nevertheless a nontrivial and challenging task, and it is often addressed by using amphiphilic compounds as nanosized delivery vehicles. Pluronics belong to a peculiar class of biocompatible and thermosensitive nonionic amphiphilic copolymers, and their self-assemblies are employed as drug delivery excipients because of their unique properties. We herein report on the encapsulation of diclofenac sodium within Pluronic F68 self-assemblies in water, underpinning the impact of the drug on the rheological and microstructural evolution of pluronic-based systems. The self-assembly and thermoresponsive micellization were studied through isothermal steady rheological experiments at different temperatures on samples containing 45 wt % Pluronic F68 and different amounts of diclofenac sodium. The adoption of scattering techniques, small-angle X-ray scattering (SAXS) and small-angle neutron scattering (SANS), allowed for the description of the system features at the nanometer length scale, providing information about the characteristic size of each part of the micellar structures as a function of temperature and drug concentration. Diclofenac sodium is not a good fellow for Pluronic F68. The triblock copolymer aids the encapsulation of the drug, highly improving its water solubility, whereas diclofenac sodium somehow hinders Pluronic self-assembly. By using a simple empirical model and no fitting parameters, the steady viscosity can be predicted, although qualitatively, through the volume fraction of the micelles extracted through scattering techniques and compared to the rheological one. A tunable control of the viscous behavior of such biomedical systems may be achieved through the suitable choice of their composition.

## Introduction

Pluronics (trade name of Poloxamers) are nonionic amphiphilic copolymers, whose triblock structure features two lateral hydrophilic poly(ethylene oxide) (PEO) units surrounding a central segment of poly(propylene oxide) (PPO).^1^ They are biocompatible and thermosensitive synthetic polymers, and they can spontaneously form nanosized structures, showing a self-assembling behavior.^2−4^ As such, Pluronics own appealing properties suitable for an extremely wide variety of applications, ranging from tissue engineering and three-dimensional bioprinting to nanomedicine, pharmaceutics, and drug delivery.^5−13^ Depending on the size of PEO and PPO blocks, Pluronics are classified on the Pluronic grid,^14^ i.e., a diagram that reports the entire portfolio of Pluronic molecules as a function of the molecular weight of the PPO and the PEO content. They are identified using a descriptive alphanumeric name revealing their physical appearance at room temperature, molecular weight, and composition. Pluronics possess good solubilization properties in several polar and nonpolar solvents and, because of their self-assembly, form different phases according to the Pluronic type and concentration, solvent, and temperature.^15−21^ Pluronics in solution indeed exhibit a thermosensitive spontaneous self-assembly driven by the different solubilities of PPO and PEO units. Specifically, they can form a variety of thermodynamically stable supramolecular structures, such as micelles, reverse micelles, and lyotropic liquid crystals.^22,23^

Pluronic micellization, the organization of Pluronic unimers into micellar structures, has been extensively studied in aqueous solutions. A typical Pluronic micelle in water is an aggregate with the hydrophilic PEO units in contact with the solvent, sequestering the hydrophobic PPO in the micellar core. Micellization, which is a reversible process, arises at temperatures and Pluronic concentrations above a critical micellar temperature (CMT) and a critical micellar concentration (CMC). The increase in temperature drives micelle formation, determining a reduced solubility of the polymer units, particularly the PPO, which separates from the water environment and settles in the micelle center.^24^

Owing to their versatility and tunable properties, Pluronics are particularly attractive for drug delivery applications^25^ and, as such, they can be used as smart nanosized vehicles for navigating the drug cargo in the biological milieu.^26^ The interest in using Pluronics as drug delivery vectors is not recent and persists in emerging research works. For example, Chen et al.^27^ used Pluronic F68 to enhance the bioavailability and dissolution of ABT-963, a poorly soluble compound advocated for the treatment of pain and inflammation. They adopted different techniques, including differential scanning calorimetry, powder X-ray diffractometry, and scanning electron microscopy, to characterize the pluronic-based systems. Kadam et al.^28^ studied the solubilization of carbamazepine, an anticonvulsant and antiepileptic drug, in Pluronic micelles, investigating the drug effect on the micellar aggregates of different Pluronic solutions. Specifically, they analyzed aqueous solutions of Pluronics P103, P123, P84, and F127, and they used dynamic light scattering (DLS) to evaluate the characteristic size of micellar structures. With the aim to develop a promising delivery system for ophthalmic usage, Gratieri et al.^29^ combined Pluronic F127 and chitosan, obtaining a formulation with improved mechanical and textural properties—high hardness and adhesiveness—and mucoadhesive ability. The effect of pH conditions on the structural characteristics and micellization process of Pluronics P103, P123, and L43 in the presence of flurbiprofen were studied by Alexander et al.^30^ They adopted SANS, pulsed-field gradient stimulated-echo nuclear magnetic resonance, and surface tension measurements to observe the drug influence on Pluronics aggregation, with varying pH, discovering that the presence of drug generates a pH-dependent aggregation behavior. Basak and Bandyopadhyay^31^ examined the effects of ibuprofen, aspirin, and erythromycin on the shape and the size distribution of Pluronic F127 micelles by using cryo-scanning electron microscopy and DLS and studied the influence of drug hydrophobicity, temperature, and pH. Raval et al.^32^ presented a systematic characterization of the micellar behavior of pluronic-based systems by means of UV–visible spectroscopy, high-performance liquid chromatography, and DLS. Wei et al.^33^ analyzed the interaction between Pluronic F127 and hydrophilically modified ibuprofen through the combination of different techniques—such as rheology, SAXS, and nuclear magnetic resonance—and examined the influence of the drug on the transition properties of Pluronic F127. The thermosensitive solubilization properties of lamotrigine, a hydrophobic antiepileptic drug, in five different Pluronics (P84, P85, F127, F108, and F68) were studied by Singla et al.,^34^ who found that drug solubilization in pluronic micelles increases with increasing temperature. They used scattering methodologies to examine the dependence of the system morphology and structure on the temperature rise. Bayati et al.^35,36^ adopted different methodologies (e.g., SAXS and SANS) to investigate the system formed by Pluronic P123 and sodium glycodeoxycholate in water, studying the interaction between the P123 and the anionic bile salt. In addition, they studied the influence of the bile salt on the P123 self-assembly in water, investigating the potentiality of P123 of being a sequestrant of bile salts in the biological environment. Tasca et al.^37^ studied Pluronic F127 with sodium cholate as a carrier for doxorubicin hydrochloride, a biomedical formulation suitable for cancer therapy.

Within this rich pool of amphiphilic biocompatible copolymers, Pluronic F68—also known as Poloxamer 188—has been known since the 1950s.^38^ Over the years, several investigations have confirmed its potential in therapeutic applications.^39^ Pluronic F68 aqueous solutions experience a reverse thermal crystallization with increasing temperature.^15,21,40^ The phase diagram of Pluronic F68 in water discloses the existence of three distinct phases: individual unimers, disordered spherical micelles, and a body-centered cubic (BCC) crystalline phase. Beneath the CMT/CMC, the polymer exists in solution in the form of individual single unimers. At temperatures and concentrations above the CMT/CMC, the single amphiphilic chains aggregate and organize into spherical micellar structures. As far as the micellization process is concerned, the aggregation is driven by the dehydration of the hydrophobic PPO segment, which progressively reduces its solubility as the polymer concentration or temperature increases. Consequently, the aggregation of various unimers arises in order to minimize the interactions between PPO and the solvent, resulting in supramolecular micellar aggregates with the core occupied by the insoluble PPO and the shell formed by the soluble PEO blocks. A further increase of the temperature determines a change in the system microstructure and generates a swift growth of the solution viscosity. The micelles, hence, act as hard bodies, and the Pluronic system turns into a soft solid, describing a reversible, temperature-dependent liquid-to-solid transition.

In this work, we report on the mutual interactions between Pluronic F68 and diclofenac sodium, a nonsteroidal anti-inflammatory drug advocated for relieving pain and reducing inflammation^41^ that possesses good potentiality for drug delivery usage.^42−44^ Rheological and scattering measurements were performed in various aqueous solutions of Pluronic F68 and diclofenac sodium, and a qualitative and quantitative description of both macroscopic flow properties and the incoming microstructure was attempted. Specifically, we adopted rheology, SAXS, and SANS to investigate the effect of diclofenac sodium on the aggregation properties of Pluronic F68 in water, shedding light on the morphological features of the supramolecular micellar structures. We studied the micellization process of aqueous solutions with 45 wt % Pluronic F68 and various amounts of diclofenac sodium. We performed isothermal steady-state measurements at different temperatures to follow the morphological transition from unimers to spherical micelles as a function of the diclofenac sodium concentration. Small-angle scattering (X-ray and neutron) was used for accessing the organization of materials at the nanometer length scale and extracting the characteristic size of the hydrophobic and hydrophilic parts of the micelles as a function of temperature and drug content. The comparison of the rheological properties with microstructural information allowed for a consistent description of the biomedical systems, which may act as nanocarriers for the storage and then delivery of a pharmaceutical compound.

## Materials and Methods

### Materials

Aqueous solutions of Pluronic F68 (Sigma-Aldrich, St. Louis, MO) and diclofenac sodium (Tokyo Chemical Industry Co., TYO, JP) were prepared at room temperature with magnetic stirring. First, water solutions of Pluronic F68 were prepared by dispersing the polymer in cold water,^45^ and stored at 5 °C for 10 days, to allow for their dissolution. Afterward, diclofenac sodium was added to the solutions. The samples were constantly stirred until homogeneous solutions. We prepared different aqueous solutions with 45 wt % Pluronic F68 and various amounts of diclofenac sodium, ranging between 15 and 300 mM. The code F68 indicates a molecular mass of the hydrophobic PPO central blocks of ≈1700 Da, and a molecular mass of the attached PEO tails of 6700 Da, corresponding to a total PEO amount of ≈80 wt %. Diclofenac sodium is an ionic salt with a high potential to establish significant interactions with the hydrophilic PEO blocks. The samples did not show turbidity in the whole investigated temperature range, suggesting that diclofenac sodium is stable in the Pluronic F68 solution also at high concentrations^46^ (about 5 times its solubility limit in water^47^).

### Experiments

#### Rheology

Steady shear flow experiments were performed by using a rotational stress-controlled rheometer (MCR702, Anton Paar GmbH, Graz, Austria) equipped with Couette geometry. The temperature was controlled by a Peltier unit. Flow curves were obtained in a range of shear rates between 100 and 1 s^–1^, at different temperatures. During all rheological tests, samples were surrounded by a low viscous silicone oil to prevent water evaporation.

#### SAXS

Small-angle X-ray scattering measurements were performed using a Kratky compact camera SAXSess (Anton Paar GmbH, Graz, Austria) in the slit collimation configuration with Cu Kα radiation (wavelength λ = 0.15418 nm). Data were collected on a BAS-MS imaging plate (FUJIFILM) and processed using a PerkinElmer Cyclone Plus digital imaging reader. A paste cell covered with an out-of-focus Kapton film was used as a sample holder for all SAXS measurements. Data reduction was performed by extracting one-dimensional SAXS profiles as a function of *q* (=4π sin θ/λ, with θ half the scattering angle) from two-dimensional images and successive subtraction of the dark current and the empty sample holder. The so-obtained experimental (slit-smeared) SAXS profiles were then de-smeared through numerical deconvolution with the intensity curve of the primary beam, operating with the software SAXSquant 2.0, in the infinite slit approximation. The temperature was guaranteed by a temperature control unit (TCU 50) within ±0.1 °C. Before each measurement, the samples were equilibrated at the selected temperatures (20, 25, and 30 °C) for 20 min. The complete protocol is reported in the Supporting Information.

SAXS (de-smeared) data fit was performed by means of the SasView Package. In order to reduce the number of fitting parameters to a minimum, the Pluronic F68/diclofenac sodium solutions were modeled as a collection of monodisperse and homogeneous spheres (form factor), interacting via a hard-sphere potential (structure factor).^48^ It was verified that by using a more specific structure factor to account for the possible effect of diclofenac sodium on the charge of the micellar aggregates, as for instance the Hayter–Penfold Rescaled Mean Spherical Approximation (RMSA) structure factor for charged spheres,^49^ the results of the fitting procedure are like those obtained by using the selected hard-sphere structure factor with Percus–Yevick closure.^50^ As an example, we report in the Supporting Information the results of the fitting procedures using both structure factors for the sample with 75 mM diclofenac sodium.

SAXS experimental results refer to samples prepared with distilled water. However, we also studied the SAXS intensity profiles of the system without diclofenac sodium dissolved in deuterium oxide (D_2_O), with the aim of confirming that deuterated water does not affect the characteristic sizes of the spherical micelles (see the Supporting Information).

#### SANS

Experiments were performed at the Swiss Spallation Neutron Source, SINQ, Paul Scherrer Institut using a SANS-I instrument. The neutron wavelength was set to 5 Å. Sample-to-detector distances of 1.6, 4.5, and 18 m attained a broad range of scattering vectors *q*. The solutions were measured in quartz cuvettes with a thickness of 1 mm. The temperature was controlled with a Haake cooler and a sample environment that enables fine control and measurement of the temperature at the sample position. SANS experiments were performed at 5, 15, and 25 °C. The absolute intensity of the scattering curves was corrected for incoherent scattering of water, sample thickness, and transmission. The data were merged, and the model fitting was performed with the spherical form factor and the hard-sphere structure factor with SasView software.

## Results

### Rheology

**Figure 1:**
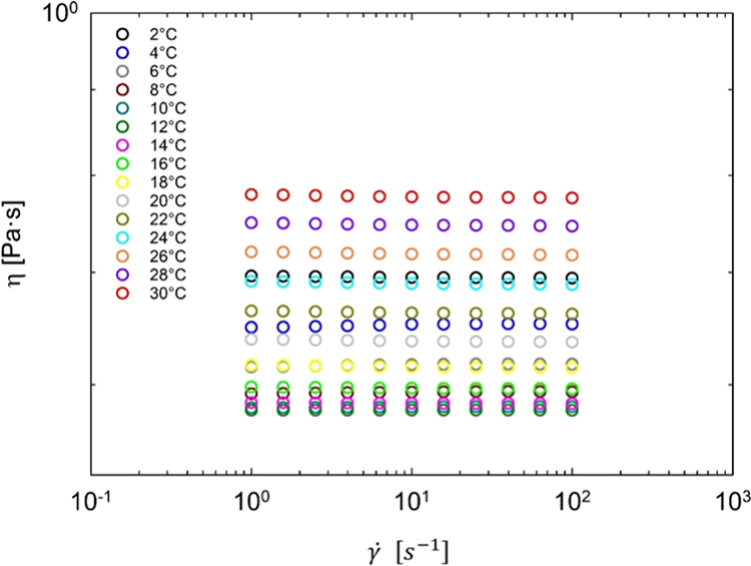
Isothermal steady measurements at different temperatures for the 45 wt % Pluronic F68 water solution with 100 mM diclofenac sodium.

We experimentally studied the rheological and phase behavior of aqueous solutions containing 45 wt % Pluronic F68 and various amounts of diclofenac sodium. Specifically, steady measurements were performed on the samples in order to obtain their isothermal flow curves at different temperatures. In Figure 1, the steady shear viscosity of the Pluronic solution with 100 mM diclofenac sodium is reported as a function of shear rate at different temperatures. Tests were conducted between 2 and 30 °C. The pluronic/drug aqueous solution is Newtonian over the entire explored temperature range.

**Figure 2:**
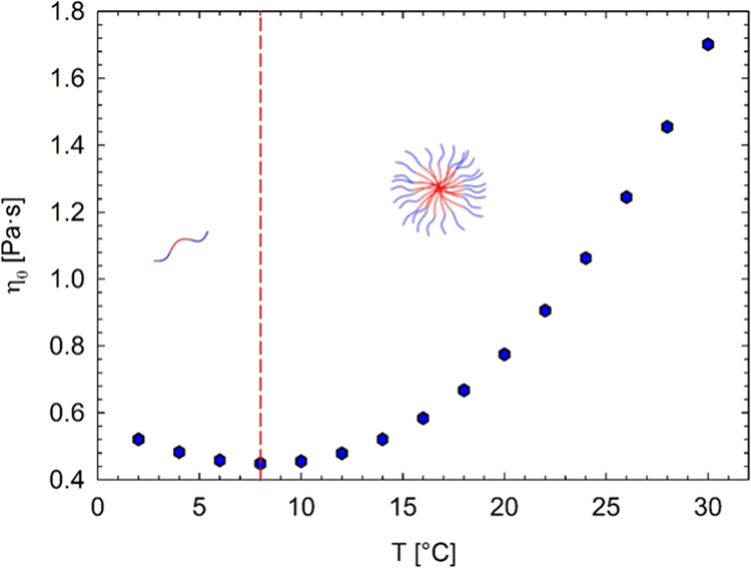
Zero-shear viscosity as a function of temperature for the 45 wt % Pluronic F68 water solution without diclofenac sodium (data obtained as linear regression of flow curves). The red dashed line indicates the onset of the micellization temperature. Unimers and micelle sketches are drawn.

The measurement of the steady viscosity vs temperature of a system experiencing a phase transition allows one to analyze its microstructural evolution at equilibrium, i.e., avoiding the dependence on thermal history kinetics. Figure 2 illustrates the variation of the steady viscosity, η_0_, with the temperature for the sample without diclofenac sodium.

As reported by Costanzo et al.,^21^ the rheological evolution of the Pluronic solution (without drug) between 2 and 30 °C is related to the transition from single unimers to spherical micelles, which occurs close to 8 °C: the steady viscosity minimum (indicated by the red dashed line in Figure 2) denotes this transition. Beneath it, we deal with aqueous solutions made by single Pluronic unimers, and η_0_ marginally depends on temperature. In particular, as expected, it decreases with increasing temperature. Above 8 °C, Pluronic unimers start to arrange themselves into spherical micellar structures, and as a consequence, η_0_ sharply increases with increasing temperature. Costanzo et al.^21^ clearly describe the increase in viscosity with temperature as mainly due to the increase in the volume fraction of the spherical micelles upon increasing temperature, in turn related to an increase in micelles number, being the micellar radius (and, as such, its volume) practically constant with temperature.

**Figure 3:**
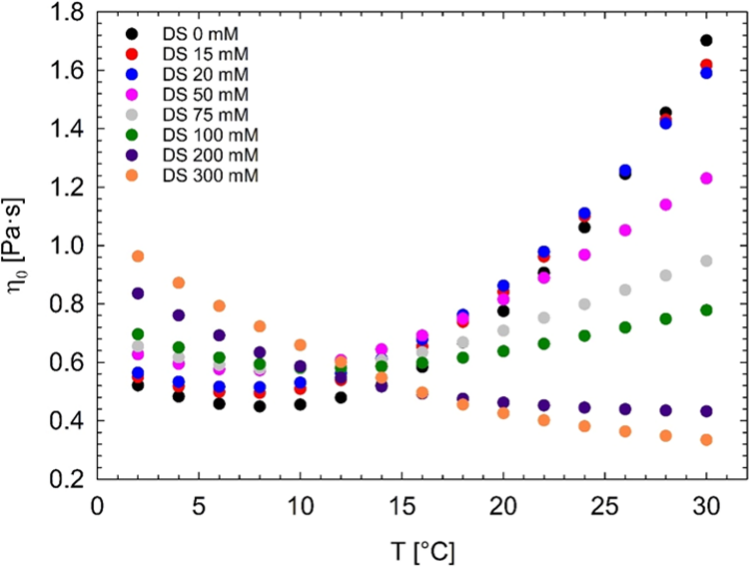
Zero-shear viscosity as a function of the temperature of the investigated samples (data obtained as linear regression of flow curves). DS stands for diclofenac sodium.

Figure 3 illustrates the variation of the steady viscosity, η_0_, with the temperature for all investigated samples.

**Figure 4:**
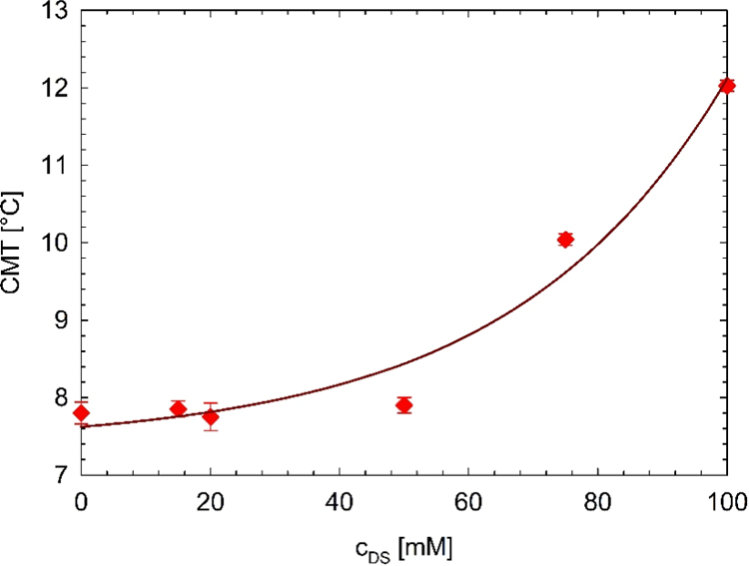
Critical micellar temperature as a function of the diclofenac sodium concentration. The solid dark red line is the exponential growth regression (eq 1). The error bars are evaluated as the standard deviations of multiple experiments.

The Pluronic solutions with a diclofenac sodium content up to 100 mM present a nonmonotonic trend with temperature similar to that of the sample with no drug. In particular, the solution with 100 mM diclofenac sodium shows a viscosity minimum at roughly 12 °C. The position of the minimum is only slightly shifted to a higher temperature, suggesting that the (limited) presence of the drug has only a minor effect in delaying the self-assembly process. As regards the Pluronic samples with 200 and 300 mM diclofenac sodium, the zero-shear viscosity has no minimum, supporting the idea of a dearth of spherical micelles or a modest development of micellar structure. Figure 4 shows the evolution of the CMT as a function of diclofenac sodium concentration (*c*_DS_).

The experimental critical micellization temperatures as a function of diclofenac sodium concentration reported in Figure 4 can be fitted by an exponential function as follows:
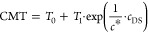
1with three fitting parameters, namely, *T*_0_, *T*_1_, and c*, equal to 7.4 ± 0.5 °C, 0.23 ± 0.26 °C, and 33 ± 0.01 mM, respectively. Equation 1 represents an example of a simple predictive tool for evaluating the CMT of these drug delivery systems. As shown in Figures 3 and 4, the CMT can be defined only for the Pluronic systems with a diclofenac sodium content up to 100 mM. The samples with a higher diclofenac sodium content, indeed, do not undergo a thermal micellization. The equation suggests that the presence of the drug “disactivates” the micellization process of the Pluronic molecules.

**Figure 5:**
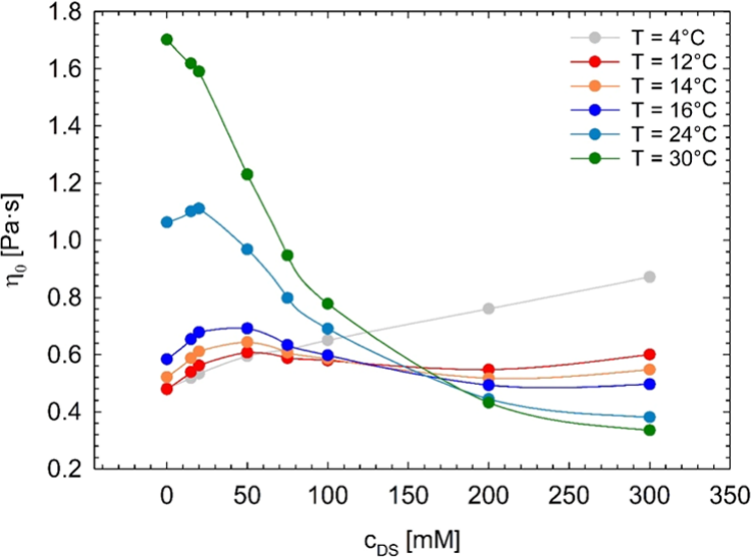
Zero-shear viscosity as a function of diclofenac sodium concentration at different temperatures, as indicated by the legend.

It is worth plotting the zero-shear viscosity as a function of the diclofenac sodium concentration and temperature, as shown in Figure 5. Clearly, the reported trends are not trivial and suggest that the combination of concentration and temperature intriguingly affects the macroscopic response of the systems, and, hence, their microstructure.

The trend described in Figure 5 denotes the absence, at the lowest and highest temperatures, of a zero-shear viscosity peak, which instead appears in the intermediate temperature range. At low temperatures, η_0_ increases linearly with increasing diclofenac sodium concentration, showing a monotonic evolution, suggesting that the drug content increase enhances the viscosity of the suspending medium where Pluronic unimers are dispersed. In other words, at low temperatures, diclofenac sodium is a sort of solution thickener. In the temperature range 12–16 °C, the trend of the zero-shear viscosity becomes nonmonotonic, revealing a peak at a value of *c*_DS_ equal to 50 mM. The increase in temperature causes the shift of the peak at lower values of *c*_DS_. At 30 °C, the peak vanishes and η_0_(*c*_DS_) is now monotonically decreasing.

While at low *c*_DS_ the effect of temperature rise is a growth of the system viscosity, as c_DS_ increases, this trend is reversed and the system becomes less viscous with increasing temperature. This is consistent with the absence of a phase transition at high *c*_DS_ values.

### SAXS

SAXS measurements were performed on aqueous solutions containing 45 wt % Pluronic F68 and various amounts of diclofenac sodium, as previously reported. We carried out experiments at three different temperatures, namely, 20, 25, and 30 °C, in order to study the system’s microstructural evolution over micellization. Furthermore, we examined the dependence of the system morphology on drug concentration. In fact, the SAXS profiles of the pluronic/drug samples possess a trend strictly dependent on the temperature and drug concentration.

**Figure 6:**
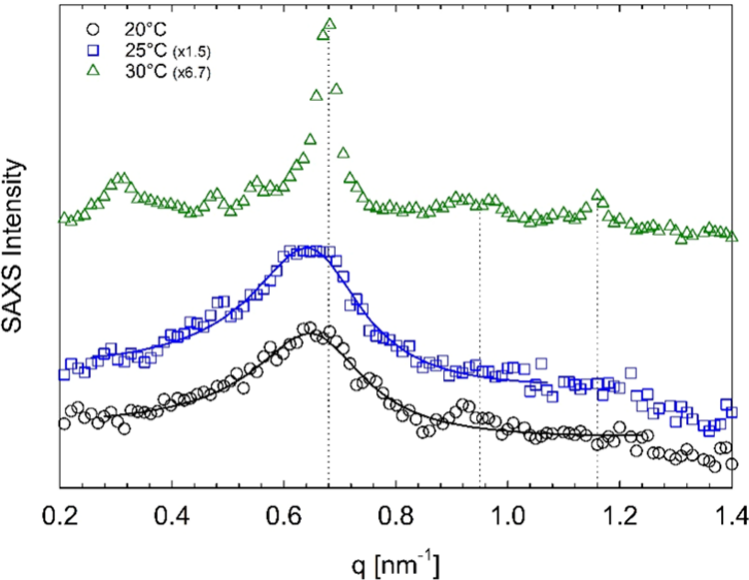
SAXS intensity profiles of 45 wt % Pluronic F68 in water with 20 mM diclofenac sodium at different temperatures, as indicated by the legend. Solid lines represent the fit to the data with the spherical form factor and the hard-sphere structure factor. The dotted vertical lines indicate the position of the Bragg peaks at q*, √2q*, and √3q* (q* ≈ 0.68 nm^–1^). Curves are vertically shifted for better visualization. The SAXS intensity is in arbitrary units. The peaks at *q* < 0.6 nm^–1^ observed for the SAXS profile collected at 30 °C are due to the formation of F68 superstructures in water, probably cubosomes.

As an example, the SAXS profiles of the Pluronic system with 20 mM diclofenac sodium measured at different temperatures are shown in Figure 6. At 20 and 25 °C, the presence of a broad correlation peak implies the existence of disordered micelles. The ordering process and the formation of the BCC phase is instead observed at 30 °C. The SAXS profile at 30 °C shows, indeed, Bragg peaks at q*, √2q*, and √3q* (q* ≈ 0.68 nm^–1^), which is indicative of structural organization into a body-centered cubic lattice. The rearrangement of the spherical micelles in the BCC phase with increasing temperature is consistent with literature data on Pluronic F68 in water.^21,37^ The SAXS profile of Figure 6 collected at 30 °C also shows the presence of additional peaks at *q* ≈ 0.3, 0.46, and 0.52 nm^–1^. These peaks are probably due to the organization of portion of F68 chains in more complex superstructures such as cubosomes,^51^ coexisting with the BCC phase. Similar SAXS patterns, which show an ordering of the micellar structures with increasing temperature, are also observed for both the systems without drug and the one with 15 mM diclofenac sodium (see Figure 9). It is worth noting that the cubosome formation was recently achieved by subjecting F68/water mixtures at F68 concentrations between 45 and 60% to slow stirring at ambient temperature, for long times (at least 14 days).^51^ The reason underlying the simultaneous formation of a BCC phase and cubosomes in our systems at 30 °C may be ascribed to the adoption of different preparation conditions, involving the storage at 5 °C for the F68/water mixtures for long times (see the Materials and Methodssection).

**Figure 7:**
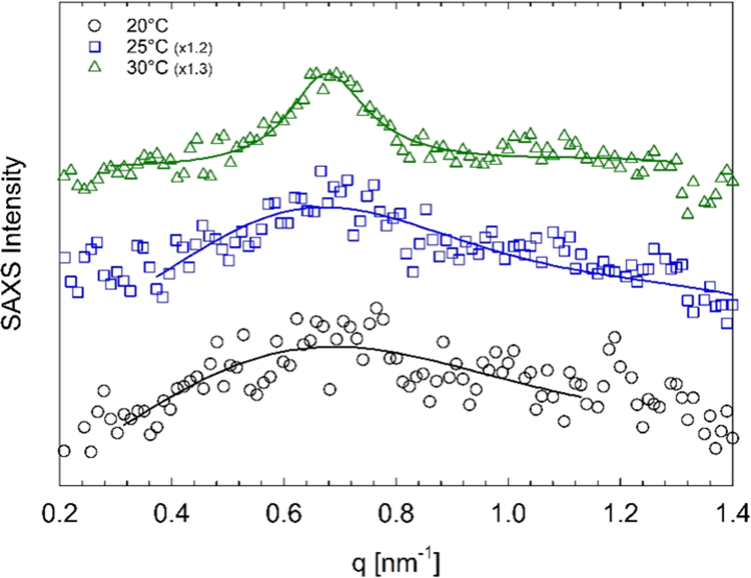
SAXS intensity profiles of 45 wt % Pluronic F68 in water with 75 mM diclofenac sodium at different temperatures, as indicated by the legend. Solid lines represent the fit to the data with the spherical form factor and the hard-sphere structure factor. Curves are vertically shifted for better visualization. The SAXS intensity is in arbitrary units.

The Pluronic systems with a diclofenac sodium content between 50 and 100 mM exhibit a micellization process that, in the temperature range 20–30 °C, does not culminate in a BCC phase organization. Their SAXS profiles recorded at 20, 25, and 30 °C all have a single broad correlation peak at a low *q*, indicating the presence of a disordered micellar system. We show in Figure 7 the SAXS profiles of the 45 wt % Pluronic sample with 75 mM diclofenac sodium recorded at different temperatures.

**Figure 8:**
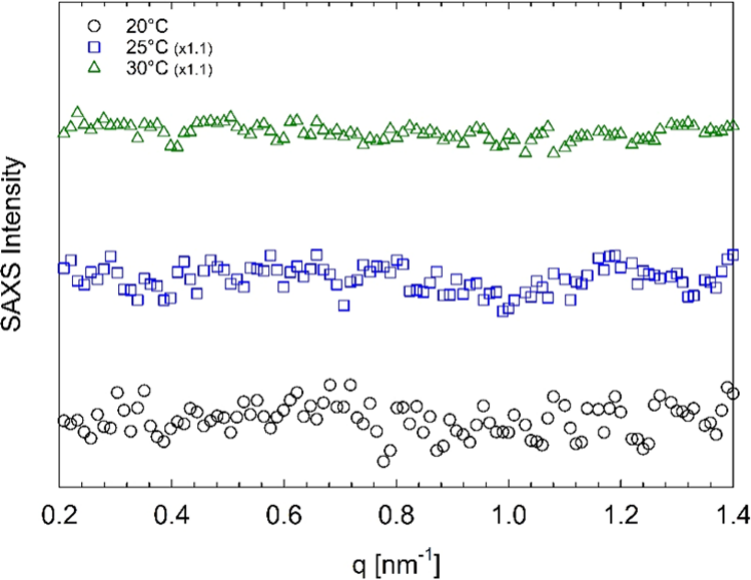
SAXS intensity profiles of 45 wt % Pluronic F68 in water with 300 mM diclofenac sodium at different temperatures, as indicated by the legend. Curves are vertically shifted for better visualization. The SAXS intensity is in arbitrary units.

The Pluronic systems with 200 mM and 300 mM diclofenac sodium unveil a completely different scenario.^46^ Their SAXS profiles (recorded at 20, 25, and 30 °C) are featureless over the entire *q* range, revealing the absence of any self-assembly phenomenon. Figure 8 illustrates the SAXS profiles of the 45 wt % Pluronic sample with 300 mM diclofenac sodium recorded at different temperatures.

**Figure 9:**
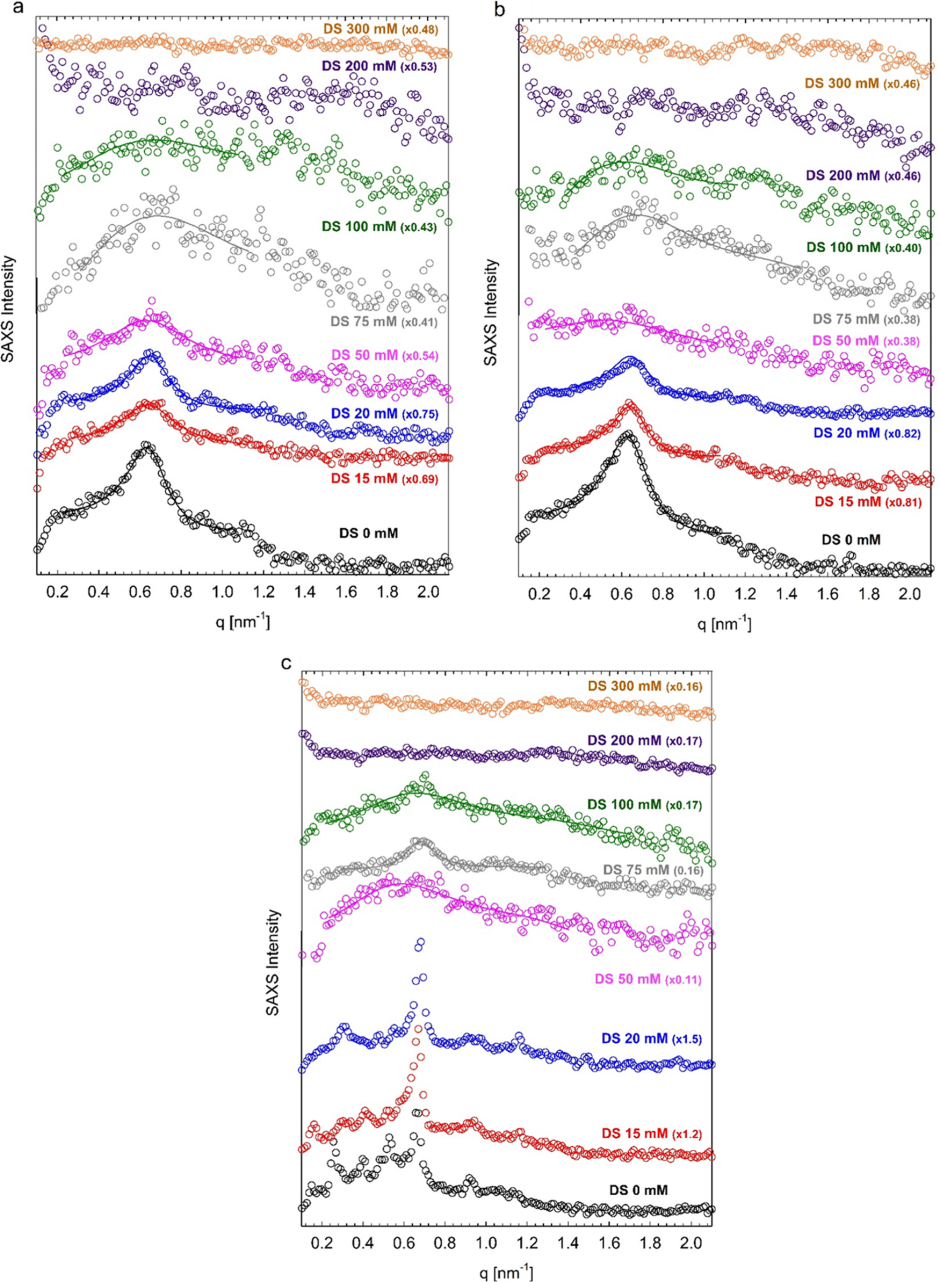
SAXS intensity profiles of 45 wt % Pluronic F68 in water with the indicated concentration of diclofenac sodium (DS) collected at 20 °C (a), 25 °C (b), and 30 °C (c). Solid lines represent the fit to the data, with the spherical form factor and the hard-sphere structure factor. Curves are vertically shifted for better visualization. The SAXS intensity is in arbitrary units. The peaks at *q* < 0.6 nm^–1^ for samples with 0, 15, and 20 mM diclofenac sodium in c are due to the formation of F68 superstructures in water, probably cubosomes.

Figure 9 summarizes the SAXS profiles of all investigated systems, highlighting their dependence on the diclofenac sodium concentration.

**Figure 10:**
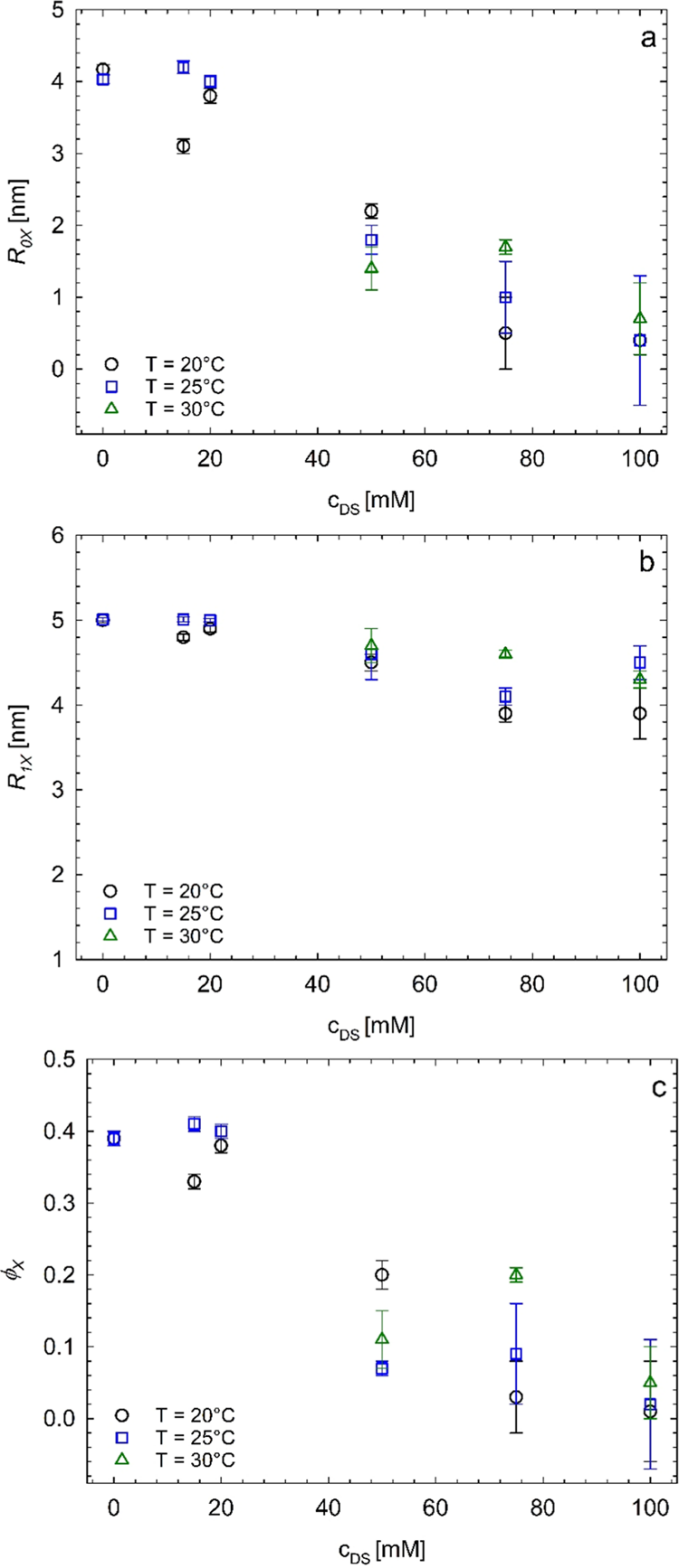
Values of the apparent core radius *R*_0*X*_ (a), the hard-sphere radius *R*_1*X*_ (b), and the volume fraction of the micellar aggregates ϕ_*X*_ (c) extracted from the fit to the SAXS profiles of Figures 6, 7, and 9, relative to the Pluronic F68/diclofenac sodium systems organized in micellar aggregates with the spherical form factor and the hard-sphere structure factor.

The fit to the SAXS profiles of the systems organized in micellar aggregates with the spherical form factor and the hard-sphere structure factor are shown in Figures 6, 7, and 9 (continuous lines). The values of the model parameters, that is, the values of the apparent core radius *R*_0*X*_, the hard-sphere radius (approximately coincident with the total micellar radius *R*_1*X*_), and the volume fraction of the micellar aggregates ϕ_*X*_, extracted from the fit, are reported in Figure 10 and Table S2.

It is apparent that in the temperature range 20–30 °C the values of *R*_0*X*_ and ϕ_*X*_ decrease upon increasing drug concentration, while *R*_*1X*_ remains almost constant. In particular, the values of *R*_*0X*_ decrease from ≈4 to ≈1 nm, and those of ϕ_*X*_ from ≈0.4 to ≈0.1, whereas the values of *R*_1*X*_ remain around 4–5 nm.

The results of Figure 10 (and of Table S2) indicate that—at low drug content—SAXS probes an apparent core of radius of ≈4 nm, only slightly lower than the micellar radius of ≈5 nm, regardless of temperature. This small difference is because the portions of PEO blocks emerging at the PPO boundary tend to form, for topological reasons, a compact corona around it with scarce or null interpenetration of water molecules, generating a low contrast in electron density with the effective PPO core. Contrast arises only at radial distances from the core higher than *R*_0*X*_, corresponding to a distance where the surface area available for hydration of PEO moieties becomes higher. For this reason, the inner core radius, *R*_0*X*_, represents the radius of the effective hydrophobic aggregates of PPO chains plus the thickness of a thin layer of the surrounding PEO chains scarcely swollen by water. This core, in turn, is surrounded by a fully hydrated shell of PEO moieties with thickness δ = *R*_1*X*_ – *R*_0*X*_.

Upon increasing the drug concentration in the tested temperature range, the favorable ionic interactions between the drug molecules and the PEO blocks increase the overall solubility of the Pluronic F68 chains (salting-in). Consequently, a neat decrease of the volume fraction of the micellar aggregates occurs because a lesser number of Pluronic F68 chains are available for hydrophobic aggregation. Simultaneously, the apparent core radius of the survival micellar aggregates decreases, probably because of the increase in the ionic strength of the environment. The radius of the micellar aggregates *R*_*1X*_, instead, is less sensitive to the presence of ionic drug molecules. At 200 mM drug concentration, the solubility of the Pluronic F68 chains in the medium is almost complete, and no micellar aggregates are formed, regardless of the temperature. Therefore, before inducing the complete destruction of the aggregation state of Pluronic F68 molecules, the progressive increase of diclofenac sodium salt concentration induces a gradual decrease of the number of Pluronic F68 chains participating in the micellar aggregates and a gradual decrease of the effective core radius of the formed micelles. Values of the core ratio close to 1 nm are reached at a drug concentration of 100 mM, consistent with a compact conformation of PPO blocks.

Therefore, while in the absence of diclofenac sodium, the effect of temperature results in a system ordering process, the addition of diclofenac sodium in the Pluronic solutions causes a progressive reduction of the system order, ruining or even banishing the micellization process. At 20 and 25 °C, the 45 wt % Pluronic system (without drug) appears as a suspension of disordered hard spheres. By increasing diclofenac sodium concentration, the system becomes increasingly disordered, and the inner core size decreases. At 30 °C, the tendency of the system to self-organize in a BCC lattice is still retained at 15 and 20 mM diclofenac sodium. However, a further increase in the drug concentration provokes the disruption of the BCC phase organization, giving rise to disordered spherical micelles. In the entire investigated temperature range, the samples with a drug content of 200 and 300 mM own a SAXS response that does not describe any ordered/disordered system.

With the aim of extending the structural characterization of the Pluronic systems by performing SANS measurements in heavy water, we have checked that the micellar parameters extracted from SAXS data collected for the Pluronic F68 dissolved in D_2_O are quantitatively similar to those extracted from SAXS data collected for Pluronic F68 dissolved in distilled water. One example is reported in Table S1, which shows that the reported results of the fitting of the SAXS data collected at 20 °C for the 45 wt % Pluronic F68 dissolved in D_2_O are quantitatively equal, within experimental error, to those obtained from SAXS measurement on the Pluronic dissolved in distilled water (Table S2).

### SANS

The structural investigation of the systems organized in micellar aggregates was further extended by collecting SANS profiles at 5, 15, and 25 °C for the 45 wt % Pluronic F68/diclofenac sodium solutions (diclofenac sodium concentration equal to 0, 20, 30, 50, 100, and 300 mM) dissolved in D_2_O. The SANS curves are shown in Figures S1 and S2. The lower temperatures probed in SANS experiments were purposely selected in order to prevent the ordering of micellar aggregates in BCC superstructures, even at low drug concentrations. Indeed, preliminary rheological measurements indicate that D_2_O may cause ordering at lower temperatures than water. The effect of D_2_O is evident also at a high concentration of diclofenac sodium, 200 and 300 mM. At these drug concentrations, we have no self-assembly in water (Figures 8 and 9), but self-assembly in D_2_O (Supporting Information) (vide infra).

**Figure 11:**
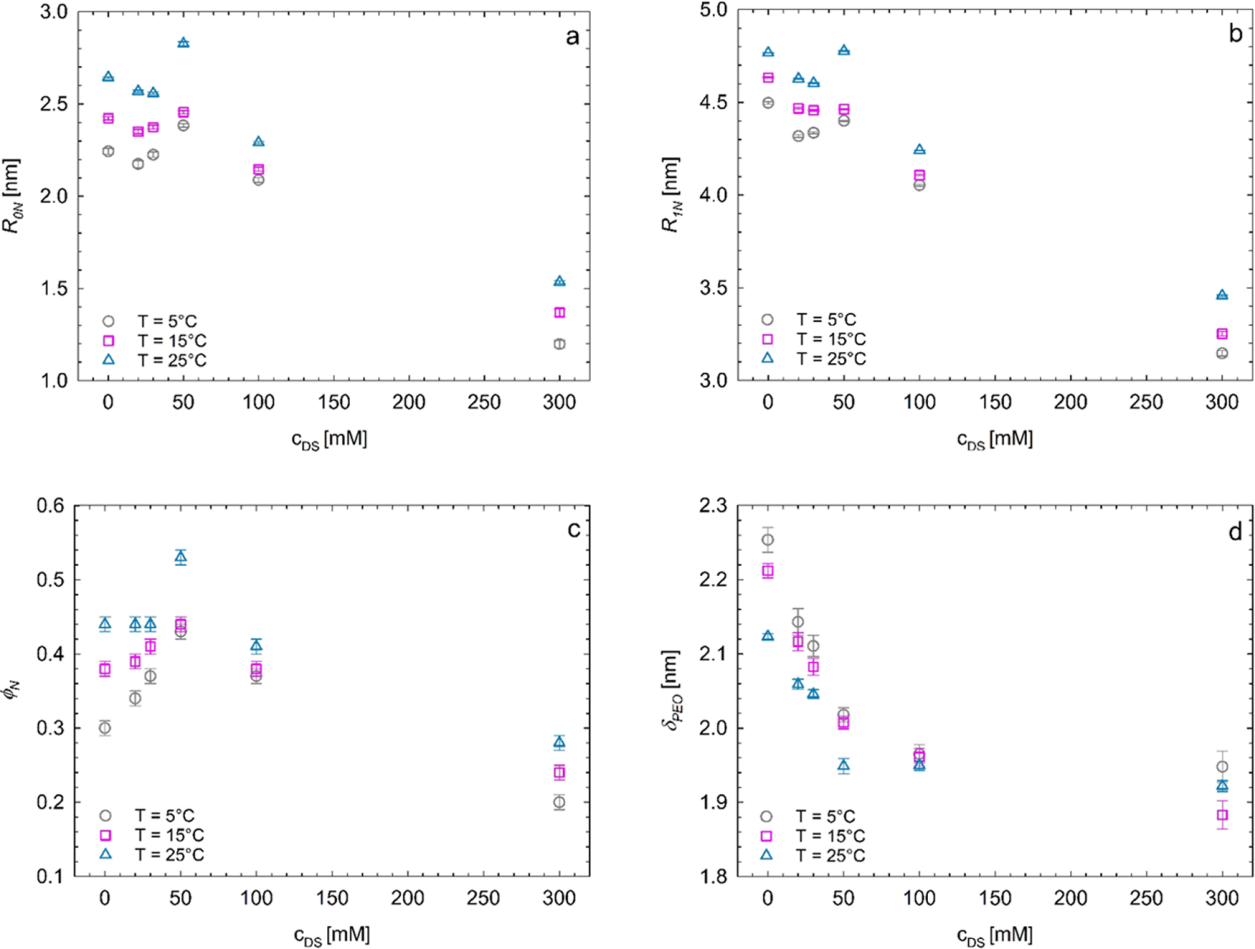
Values of the PPO core radius *R*_0*N*_ (a), the hard-sphere radius *R*_1*N*_ (b), the volume fraction of the micellar aggregates ϕ_*N*_ (c), and the thickness of PEO layer δ_PEO_ (d) extracted from the fit to the SANS data relative to the Pluronic F68/diclofenac sodium solutions in D_2_O with the spherical form factor and the hard-sphere structure factor.

The SANS curves of Figures S1 and S2 show a well-defined correlation peak, regardless of temperature, similar to the SAXS profiles in Figures 6, 7, and 9, relative to the systems organized in micellar aggregates. By fitting the SANS data with the spherical form factor and the hard-sphere structure factor, the values of the inner core radius *R*_0*N*_, hard spheres radius *R*_1*N*_, and volume fraction ϕ_*N*_ were extracted. The so-obtained parameters are listed in Table S3 and Figure 11. The values of the PEO thickness of the spherical micelles, δ_PEO_ = *R*_1*N*_ – *R*_0*N*_, are also reported in Figure 11.

It is apparent that while the values of the hard-sphere radius and micellar volume fraction extracted from SAXS (*R*_1*X*_ and ϕ_*X*_, respectively) and SANS (*R*_1*N*_ and ϕ_*N*_, respectively) data analysis are similar, the inner core radii probed by neutrons (*R*_0*N*_) are smaller than those probed by X-rays (*R*_0*X*_). More specifically, both techniques indicate that the inner core radius decreases with increasing drug concentration. However, while SAXS measurements show a decrease between about 4 and 1 nm, SANS measures a decrease from 3 to 1 nm, roughly.

The difference in the values of the inner core radius seen by SAXS and SANS can be attributed to the different contrasts seen by the two probes. Differences, in contrast, arise because water swells the PEO chains of the Pluronic molecules, but the corona immediately adjacent to the hydrophobic PPO core is probably less swollen for topological reasons. Therefore, as already discussed before, X-rays do not probe the effective radius of the PPO core but a larger core radius, containing also a scarcely swollen PEO layer. On the other hand, neutrons probe an enhanced contrast, due to the use of D_2_O, so that the PEO corona immediately positioned around the PPO core and the core itself can be well distinguished, thus resulting in the “real” measurement of the PPO core.

It is worth noting that, unlike SAXS results, SANS also assesses the presence of spherical micelles for the 300 mM sample, for which the SAXS pattern previously discussed is featureless. This is probably due to the influence of deuterated water on Pluronic self-assembly, which will be the topic of a future work.

## Conclusions

The rheological and microstructural evolution of Pluronic F68 self-assemblies in water containing diclofenac sodium were investigated as a function of concentration and temperature. The micellization process was studied through rheology and complementary scattering techniques, i.e., SAXS and SANS. Macroscopic flow measurements suggested that at diclofenac sodium concentrations higher than 100 mM the drug adversely influences the formation of Pluronic spherical micelles. SAXS and SANS data revealed that the spherical micellar sizes depend on drug concentration, the increase of which causes system disorder and hinders the micellization process. Scattering measurements and modeling indicate that the volume fraction and the PPO core radius decrease with increasing diclofenac sodium concentration; the micellar radius is instead almost independent of drug concentration.

The comparison with our previous study^46^ reveals that the adoption of scattering methodologies provides crucial information about the microstructural evolution of such systems in the presence of drugs. In our previous work, we studied the same systems by employing experimental rheology and Nuclear Magnetic Resonance (NMR). We built a rheological phase diagram for 45 wt % Pluronic F68 with different amounts of diclofenac sodium in water, shedding light on the interactions between the amphiphilic copolymer and the drug. At drug concentrations above 200 mM, we did not detect any phase transition with increasing temperature. These outcomes are well supported by the current work, which unveils that in the presence of a significant amount of drug, the micellization process is hindered.

**Figure 12:**
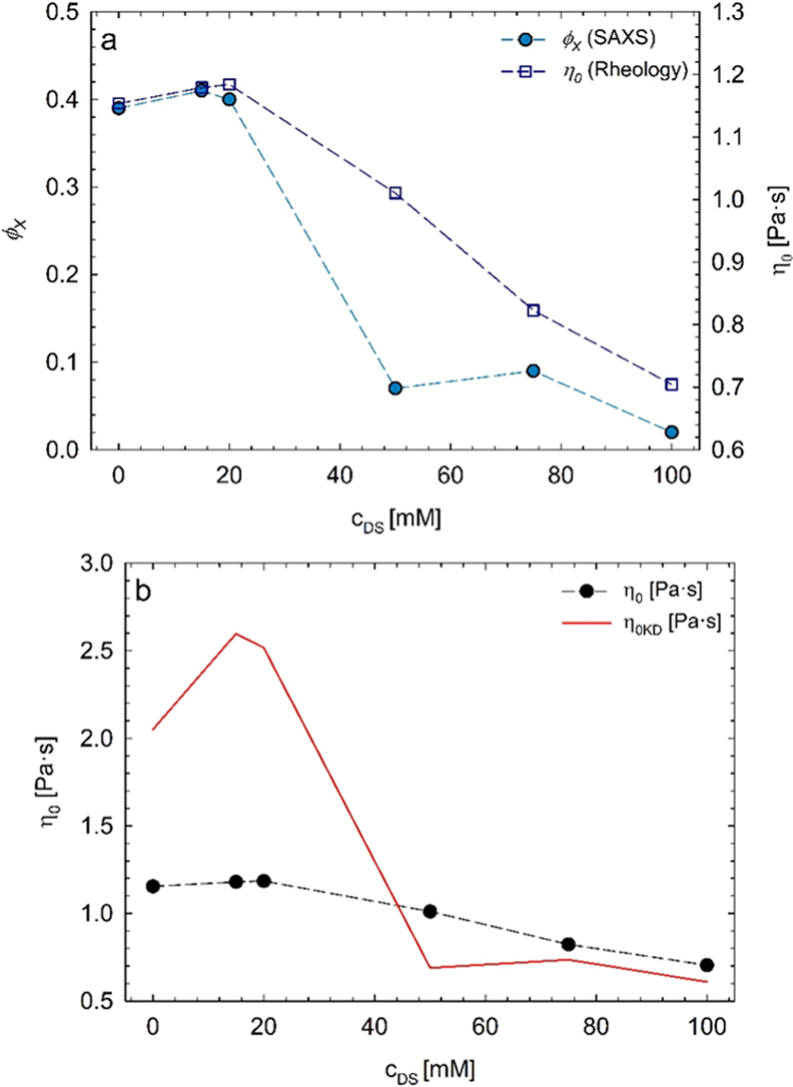
(a) Micellar volume fraction extracted from SAXS analysis (ϕ_*X*_) and zero-shear viscosity (η_0_) as a function of diclofenac sodium concentration, at 25 °C. (b) Zero-shear viscosity measured by means of rheology and zero-shear viscosity computed through the Krieger–Dougherty equation (η_0KD_).

It is worth remarking that the gradual decrease in the steady viscosity of the solutions with increasing drug content mimics the decrease in the volume fraction. As an example, Figure 12a reports the micellar volume fraction extracted from SAXS analysis (ϕ_*X*_) along with the zero-shear viscosity (η_0_) as a function of diclofenac sodium concentration, at 25 °C. The experimental trend of viscosity data can be treated with the Krieger–Dougherty equation (eq 2), which empirically describes the relation between the zero-shear viscosity and the volume fraction of hard-sphere suspensions,^21,52,53^ although noncolloidal:
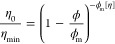
2In eq 2, ϕ is the volume fraction, ϕ_m_ is the maximum packing volume fraction of randomly distributed spheres, [η] is the intrinsic viscosity, and η_min_ is the level of viscosity above which the transition from unimers to spherical Pluronic aggregates occurs. The value of ϕ_m_ strongly depends on the particle size distribution and increases with increasing polydispersity. For a system of monodisperse hard spheres,^53^ ϕ_m_ ≈ 0.63–0.64, and [η] ≈ 2.5. Equation 2 has been successfully used in describing the dependence of the viscosity on the volume fraction of Pluronic spherical self-assemblies.^21^

Figure 12b shows the comparison between the steady viscosity, measured by means of rheology (macroscopic information), and the steady viscosity computed through eq 2 (η_0KD_), where the micellar volume fraction is fixed to the values extracted from SAXS data analysis (ϕ = ϕ_*X*_) (microscopic approach). The steady viscosity minimum values, η_min_, were evaluated through Figure 3, at each drug concentration. The agreement between the measured viscosity and the one extracted from the volume fraction analysis is undeniable, even more so because of no use of fitting parameters. The agreement remains qualitative, due probably to the various approximations used in eq 2 (i.e., non-Brownian suspensions, absence of polydispersity, etc.).

In conclusion, the current work allows identifying a novel potential drug delivery system, able to store huge amounts of drug molecules, and unraveling the interactions between very peculiar amphiphilic molecules and a commonly used anti-inflammatory drug.

## Supporting Information Available

Protocol for SAXS experiments; fitting parameters obtained from SAXS data analysis relative to the 45 wt % Pluronic F68/D_2_O solution without diclofenac sodium (Table S1); fitting parameters obtained from SAXS data analysis relative to the 45 wt % Pluronic F68/diclofenac sodium/water solutions (Table S2); SANS profiles for the 45 wt % Pluronic F68/D_2_O solutions with diclofenac sodium (Figures S1 and S2); fitting parameters obtained from SANS data analysis relative to the 45 wt % Pluronic F68/diclofenac sodium/D_2_O solutions (Table S3); equations for scattering data fits (eqs S1–S5); SAXS intensity profiles of 45 wt % Pluronic F68 in water with 75 mM diclofenac sodium, at 30 °C, and the fit to the data with the spherical form factor, and the hard-sphere structure factor or the Hayter–Penfold Rescaled Mean Spherical Approximation (RMSA) structure factor (Figure S3); and fitting parameters obtained from SAXS data relative to the 45 wt % Pluronic F68 in water with 75 mM diclofenac sodium, at 30 °C, with the spherical form factor, and the hard-sphere structure factor or the Hayter–Penfold Rescaled Mean Spherical Approximation (RMSA) structure factor (Table S4) (PDF)
