# Supplementary material for: Pluronic F68 Micelles as Carriers for an Anti-Inflammatory Drug: A Rheological and Scattering Investigation
Source: Langmuir. 2024 Jan 3;40(2):1544–54. doi: 10.1021/acs.langmuir.3c03682 (PMC10795184; doi:10.1021/acs.langmuir.3c03682)
Supplement: Supplementary file 1 — la3c03682_si_001.pdf [file la3c03682_si_001.pdf]

Supporting Information

Pluronic F68 micelles as carriers for an anti-inflammatory drug: a rheological and scattering investigation

Nicola Antonio Di Spirito[1], Nino Grizzuti[1], Viviane Lutz-Bueno[2], Gaia Urciuoli[3], Finizia Auriemma[3], and Rossana Pasquino[1,*]

[1] DICMaPI, Università degli Studi di Napoli Federico II, P. le Tecchio 80, 80125 Napoli, Italy

[2] Laboratory for Neutron Scattering & Imaging, Paul Scherrer Institute, CH-5232 Villigen PSI, Switzerland

[3] Dipartimento di Scienze Chimiche, Università di Napoli "Federico II", Complesso Monte S.Angelo, via Cintia, 80126 Napoli, Italy

**corresponding author: r.pasquino@unina.it*

KEYWORDS: Pluronics / Poloxamers | Diclofenac Sodium | Drug Delivery | Micelles | Rheology | Scattering | SAXS | SANS

S1. SAXS

We adopted the following protocol for each SAXS measurement:

- samples loading at 20˚C and waiting 20 min for thermal equilibrium;
- performing SAXS measurement at 20˚C for 20 min;
- heating up to 25˚C and waiting 20 min for thermal equilibrium;
- performing SAXS measurement at 25˚C for 20 min;
- heating up to 30˚C and waiting 20 min for thermal equilibrium;
- performing SAXS measurement at 30˚C for 20 min;
- cooling up to 25˚C and waiting 20 min for thermal equilibrium;
- performing further SAXS measurement at 25˚C for 20 min to check the process repeatability.

T [˚C]	Core radius, R_{0X} [nm]	Hard sphere radius, R_{1X} [nm]	Volume fraction of micellar aggregates, ϕ_X

20	4.83 ± 0.05	5.10 ± 0.02	0.43 ± 0.01

Table S1. Fitting parameters obtained from SAXS data analysis relative to the 45 wt% Pluronic F68/D_2O solution without diclofenac sodium. R_{0X}, R_{1X}, and ϕ_X represent the PPO core radius, the hard sphere radius, and the volume fraction of the micellar aggregates, respectively.

**T [˚C]**	**Diclofenac sodium concentration [mM]**	**Core radius, R_{0X} [nm]**	**Hard sphere radius, R_{1X} [nm]**	**Volume fraction of micellar aggregates, ϕ_X**
20	0	4.17 ± 0.07	5.00 ± 0.03	0.39 ± 0.01
	15	3.1 ± 0.1	4.80 ± 0.04	0.33 ± 0.01
	20	3.8 ± 0.1	4.90 ± 0.04	0.38 ± 0.01
	50	2.2 ± 0.1	4.5 ± 0.1	0.20 ± 0.02
	75	0.5 ± 0.5	3.9 ± 0.1	0.03 ± 0.05
	100	0.4 ± 0.9	3.9 ± 0.3	0.01 ± 0.07
25	0	4.02 ± 0.06	5.01 ± 0.02	0.39 ± 0.01
	15	4.20 ± 0.09	5.01 ± 0.03	0.41 ± 0.01
	20	4.00 ± 0.08	5.00 ± 0.02	0.40 ± 0.01
	50	1.8 ± 0.2	4.6 ± 0.3	0.07 ± 0.01
	75	1.0 ± 0.5	4.1 ± 0.1	0.09 ± 0.07
	100	0.4 ± 0.9	4.5 ± 0.2	0.02 ± 0.09
30	0	/	/	/
	15	/	/	/
	20	/	/	/

	50	1.4 ± 0.3	4.7 ± 0.2	0.11 ± 0.04
	75	1.7 ± 0.1	4.60 ± 0.05	0.27 ± 0.01
	100	0.7 ± 0.5	4.3 ± 0.1	0.05 ± 0.05

Table S2. Fitting parameters obtained from SAXS data analysis relative to the 45 wt% Pluronic F68/diclofenac sodium/water solutions. R_{0X}, R_{1X}, and ϕ_X represent the PPO core radius, the hard sphere radius, and the volume fraction of the micellar aggregates, respectively.

S2. SANS

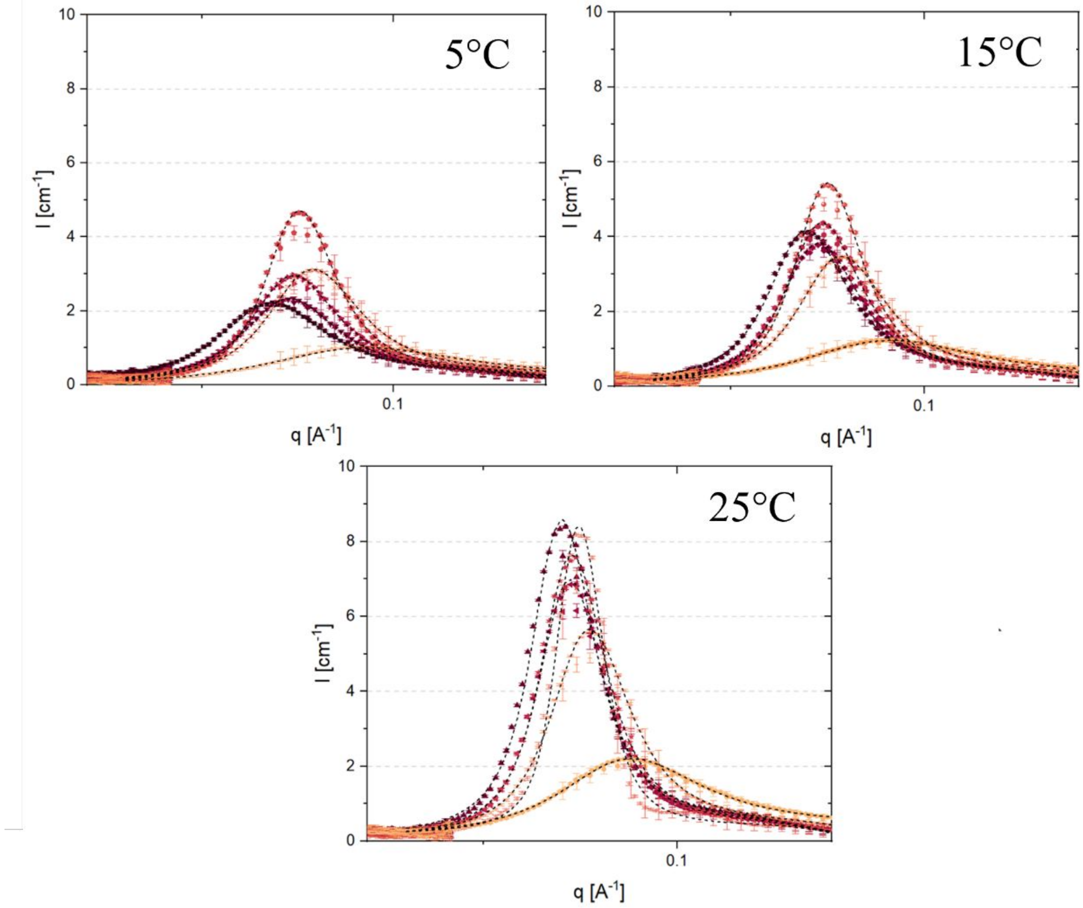

Figure S1. SANS profiles for the 45 wt% Pluronic F68/D_2O solutions with diclofenac sodium of 0, 20, 30, 50, 100 and 300 mM, collected at 5, 15, and 25˚C. Note that diclofenac sodium concentration increases from dark to light colours. The dotted lines are fits to the data with the sphere form factor and the hardsphere structure factor. Note that the peak position q^* increases with increasing the temperature.

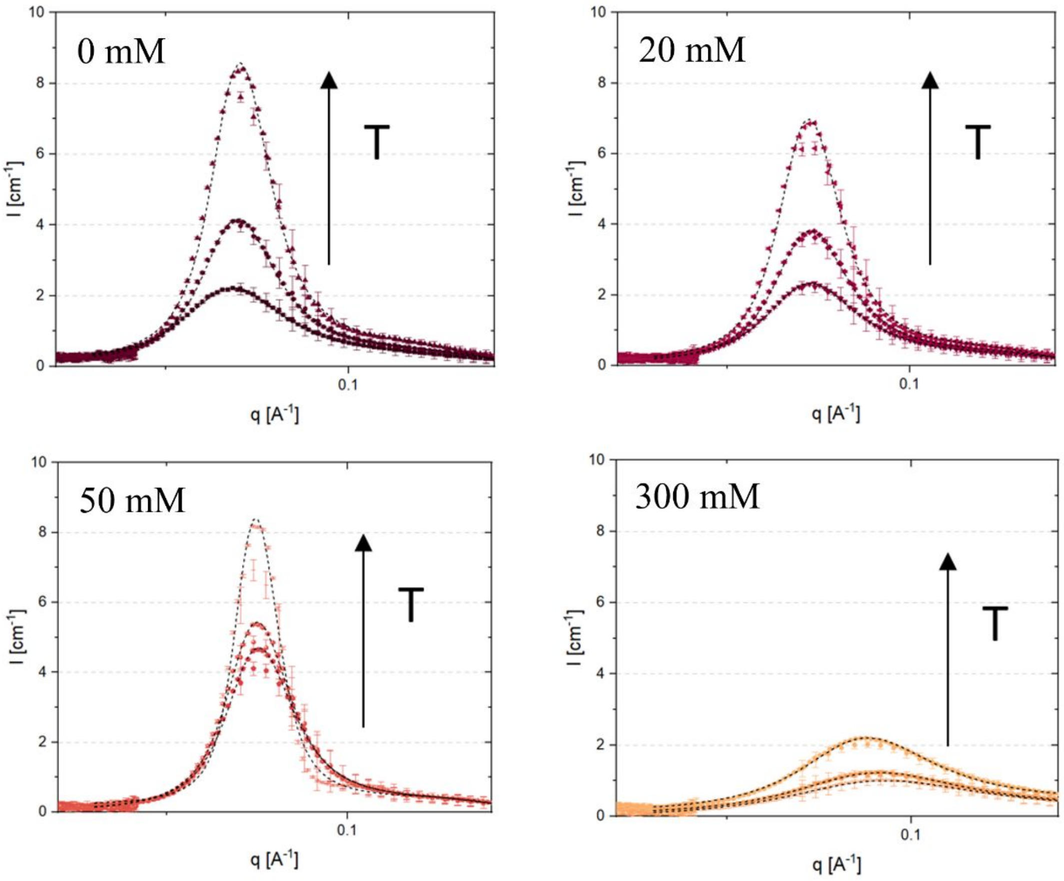

Figure S2. SANS profiles for the 45 wt% Pluronic F68/D_2O solutions with diclofenac sodium of 0 mM, 20 mM, 50 mM, and 300 mM. The dotted lines are fits with the sphere form factor and the

hardsphere structure factor. Note that with the temperature increase (T = 5, 15, and 25˚C) the peak intensity and sharpness grow.

T [˚C]	Diclofenac sodium concentration [mM]	Core radius, R_{0N} [nm]	Hard sphere radius, R_{1N} [nm]	Volume fraction of micellar aggregates, ϕ_N
5	0	2.24 ± 0.02	4.49 ± 0.01	0.30 ± 0.01
	20	2.18 ± 0.02	4.32 ± 0.01	0.34 ± 0.01
	30	2.23 ± 0.01	4.34 ± 0.01	0.37 ± 0.01
	50	2.38 ± 0.01	4.40 ± 0.01	0.43 ± 0.01
	100	2.09 ± 0.01	4.05 ± 0.01	0.37 ± 0.01
	300	1.19 ± 0.02	3.15 ± 0.01	0.20 ± 0.01
15	0	2.42 ± 0.01	4.63 ± 0.01	0.38 ± 0.01
	20	2.35 ± 0.01	4.47 ± 0.01	0.39 ± 0.01
	30	2.37 ± 0.01	4.46 ± 0.01	0.41 ± 0.01
	50	2.46 ± 0.01	4.46 ± 0.01	0.44 ± 0.01
	100	2.15 ± 0.01	4.11 ± 0.01	0.38 ± 0.01
	300	1.37 ± 0.02	3.25 ± 0.01	0.24 ± 0.01
25	0	2.64 ± 0.01	4.77 ± 0.01	0.44 ± 0.01
	20	2.57 ± 0.01	4.63 ± 0.01	0.44 ± 0.01
	30	2.56 ± 0.01	4.60 ± 0.01	0.44 ± 0.01
	50	2.83 ± 0.01	4.78 ± 0.01	0.53 ± 0.01
	100	2.29 ± 0.01	4.24 ± 0.01	0.41 ± 0.01
	300	1.54 ± 0.01	3.46 ± 0.01	0.28 ± 0.01

Table S3. Fitting parameters obtained from SANS data analysis relative to the 45 wt% Pluronic F68/diclofenac sodium/D_2O solutions. R_{0N}, R_{1N}, and ϕ_N represent the PPO core radius, the hard sphere radius, and the volume fraction of the micellar aggregates, respectively.

S3. SCATTERING DATA FITS

Scattering (desmeared) data fit was performed by means of the SasView Package, by modeling the Pluronic F68/diclofenac sodium solutions as a collection of monodisperse and homogeneous spheres (form factor, *F(q)*), interacting via a hard-spheres potential (structure factor, *S(q)*). The used models are available within the SasView Package. The theoretical expressions adopted for the investigated systems are described by the following equations:

$$F(q) = \frac{4}{3}\pi R^3 \frac{3 j_1(qR)}{qR} \tag{S1}$$

$$j_1(x) = \frac{\sin(x) - x\cos(x)}{x^2} \tag{S2}$$

$$S(q) = \frac{1}{1 + 24\phi \frac{G(qR_{HS},\phi)}{qR_{HS}}} \tag{S3}$$

$$G(v,\phi) = \frac{(1+2\phi)^2}{(1-\phi)^4 y^2}(\sin y - y\cos y) - \frac{6\phi\left(1+\frac{\phi}{2}\right)^2}{(1-\phi)^4 y^3}\left[2y\sin y + (2-y^2)\cos y - 2\right] + \frac{\phi(1}{2(1-} \left\{-y^4\cos y + 4\left[(3y^2-6)\cos y + (y^3-6y)\sin y + 6\right]\right\} \tag{S4}$$

$$\phi = \frac{4}{3}\pi \bar{R}^3 N_b \tag{S5}$$

where:

R is the sphere radius;

$j_1(x)$ is the first-order Bessel function;

ϕ is the particle volume fraction;

R_{HS} is the hard-sphere interaction radius;

$G(v,\phi)$ (with $y = qR_{HS}$) is a function given by Eq. S4;

N_b is the density number.

To reduce the number of fitting parameters to a minimum, the Pluronic F68/diclofenac sodium solutions were modeled as a collection of monodisperse and homogeneous spheres (form factor), interacting via a hard-spheres potential (structure factor). It was verified that by using a more specific

structure factor to account for the possible effect of diclofenac sodium on the charge of the micellar aggregates, as for instance the Hayter-Penfold Rescaled Mean Spherical Approximation (RMSA) structure factor for charged spheres, the results of the fitting procedure are like those obtained by using the selected hard sphere structure factor, with Percus-Yevick closure. As an example, we report the results of the fit – which accounts for charged spheres – for the sample with 75 mM diclofenac sodium. Both Figure SI3 and the relative fitting data reported in Table SI4 confirm that the hard sphere structure factor can be also used in the presence of such a small quantity of charges.

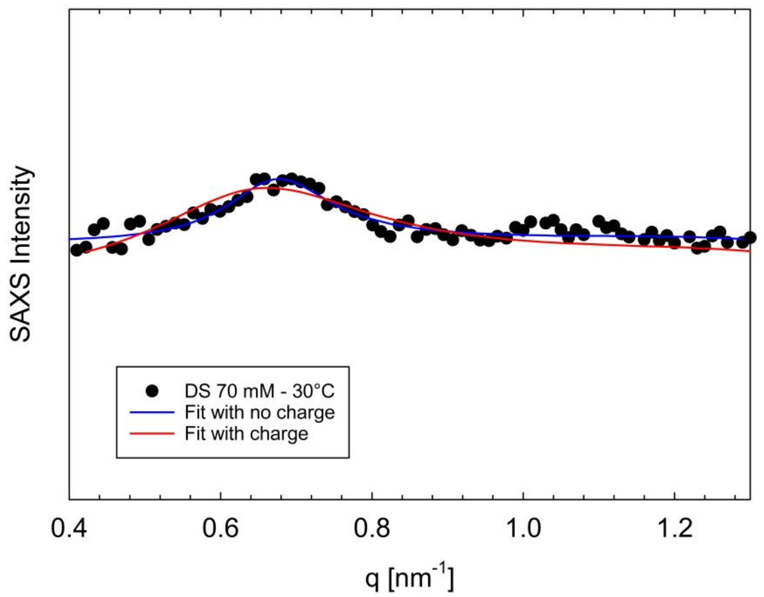

Figure S3. SAXS intensity profiles of 45 wt% Pluronic F68 in water with 75 mM diclofenac sodium, at 30˚C. Solid lines represent the fit to the data with the spherical form factor, and the hard-sphere structure factor (blue) or the Hayter-Penfold Rescaled Mean Spherical Approximation (RMSA) structure factor (red). The SAXS intensity is in arbitrary units.

Structure factor of the fitting model	Core radius, R_{0X} [nm]	Hard sphere radius, R_{1X} [nm]	Volume fraction of micellar aggregates, ϕ_X
Hard-sphere	1.7 ± 0.1	4.60 ± 0.05	0.27 ± 0.01
Hayter-Penfold Rescaled Mean Spherical Approximation (RMSA)	1.8 ± 0.1	4.54 ± 0.06	0.27 ± 0.02

Table S4. Fitting parameters obtained from SAXS data relative to the 45 wt% Pluronic F68 in water with 75 mM diclofenac sodium, at 30˚C, with the spherical form factor, and the hard-sphere structure factor or the Hayter-Penfold Rescaled Mean Spherical Approximation (RMSA) structure factor. R_{0X}, R_{1X}, and ϕ_X represent the PPO core radius, the hard sphere radius, and the volume fraction of the micellar aggregates, respectively.
